# Editor's Message

**DOI:** 10.29173/jchla29557

**Published:** 2021-04-02

**Authors:** Sandra McKeown

*[Français en suite]*

Welcome to the Spring issue of the Journal. This is the largest issue that I have been a part of to date! To that end, I would like to extend my gratitude to the other members of the editorial team as we have been busy working on a number of strategic priorities in addition to delivering the triannual publication. One of those priorities has been developing a JCHLA Data Sharing Policy, which you can read more about in one of the editorials that’s included in this issue. A huge thanks to Kevin Read at the University of Saskatchewan for leading us through this foundational work, and to the other members of the group for their valuable contributions, including Alanna Campbell, Vanessa Kitchin, Heather MacDonald, and (formerly) Erin Watson. A more recent priority for the Journal has been addressing racism and improving equity, diversity, and inclusion in the scholarly publishing process. Another editorial in this issue continues this vital conversation with our readers following the unfortunate series of events that took place with the Journal of the Medical Library Association.

We are pleased to publish the CHLA Standards for Library and Information Services in Canadian Health & Social Services Institutions 2020 in this issue. Intended to serve as a guide to structuring minimum library services within health and social services institutions across all Canadian provinces and territories, the updated Standards were made possible thanks to the tireless efforts of the CHLA-ABSC Standards Task Force (Francesca Frati, Lori Anne Oja and Julia Kleinberg) and the many individuals and groups who contributed to this important work. Well done everyone!

This is a well-rounded issue for peer-reviewed content. We have one research article, one program description, and one review article on highly relevant and timely topics such as data repositories and virtual libraries. A review article about the privacy of electronic health records comes to us from the 2020 Student Paper Prize winners, Katherine Gariépy-Saper and Nicholas Decarie. Congratulations to you both! I also wish to thank you for your patience with us as this paper is usually published in the December issue the same year it is awarded.

We also have four book reviews on topics ranging from online instruction, to library liaison programming, and managing metadata, as well as a product review for the project management solution Trello. A big thanks goes out to all of our book, product and peer reviewers for their meaningful contributions!

Sincerely,

**Sandra McKeown**

JCHLA/JABSC Editor-in-Chief

Email: editor@chla-absc.ca

Bienvenue au numéro de printemps du Journal. Il s'agit du plus gros numéro dans lequel j'ai été impliqué jusqu'à présent ! À cet égard, je tiens à exprimer ma gratitude aux autres membres de l'équipe de rédaction, car nous avons été occupés à travailler sur un certain nombre de priorités stratégiques en plus de la publication triennale. L'une de ces priorités a été l'élaboration d'une politique de partage des données du JABSC/JCHLA, que vous pouvez lire dans l'un des éditoriaux inclus dans ce numéro. Un grand merci à Kevin Read, de l'Université de Saskatchewan, pour nous avoir guidés dans ce travail fondamental, ainsi qu'aux autres membres du groupe pour leur précieuse contribution, notamment Alanna Campbell, Vanessa Kitchin, Heather MacDonald et (auparavant) Erin Watson. Une priorité plus récente de la Revue a été de s'attaquer au racisme et d'améliorer l'équité, la diversité et l'inclusion dans le processus d'édition savante. Un autre éditorial de ce numéro poursuit cette conversation essentielle avec nos lecteurs suite à la série d'événements malheureux qui se sont produits au Journal of the Medical Library Association.

Nous sommes heureux de publier dans ce numéro les Normes pour les bibliothèques et les services d’information des établissements de soins de santé et de services sociaux du Canada 2020. Destinées à servir de guide pour structurer les services de bibliothèque élémentaires au sein des établissements de santé et de services sociaux dans toutes les provinces et tous les territoires du Canada, les normes mises à jour ont été rendues possibles grâce aux efforts inlassables du groupe de travail sur les normes de l'ABSC / CHLA (Francesca Frati, Lori Anne Oja et Julia Kleinberg) et aux nombreuses personnes et groupes qui ont contribué à cet ouvrage important. Bravo à tous !

Ce numéro est bien équilibré en termes de contenu évalué par les pairs. Nous avons un article de recherche, une description de programme et un article de synthèse sur des sujets très pertinents et d'actualité tels que les dépôts de données et les bibliothèques virtuelles. Un article de synthèse sur la confidentialité des dossiers médicaux électroniques nous vient des lauréats du prix 2020 de l'article étudiant, Katherine Gariépy-Saper et Nicholas Decarie. Félicitations à vous deux ! Je tiens également à vous remercier de votre patience, car cet article est généralement publié dans le numéro de décembre de l'année où le prix est décerné.

Nous avons également quatre critiques de livres sur des sujets allant de l'enseignement en ligne à la programmation des liaisons de bibliothèques, en passant par la gestion des métadonnées, ainsi qu'une critique de produit pour la solution de gestion de projet Trello. Nous tenons à remercier tous nos évaluateurs de livres, de produits et de pairs pour leurs contributions significatives !

Sincèrement,

**Sandra McKeown**

JCHLA/JABSC Rédactrice en chef

Courriel: editor@chla-absc.ca

